# Quyu Shengxin Decoction Alleviates DSS-Induced Ulcerative Colitis in Mice by Suppressing RIP1/RIP3/NLRP3 Signalling

**DOI:** 10.1155/2021/6682233

**Published:** 2021-08-20

**Authors:** Chuang Wu, Haojie Yang, Changpeng Han, Qingming Wang, Haiyan Zhang, Ting Huang, Wenjing Mao, Cheng Tang, Wenjun Zhao, Zhiming Zhu, Jing Xu, Wei Yang

**Affiliations:** ^1^Shanghai Baoshan District Hospital of Integrated Traditional Chinese and Western Medicine, Shanghai 201999, China; ^2^Yueyang Hospital of Integrated Traditional Chinese and Western Medicine, Shanghai University of Traditional Chinese Medicine, Shanghai 200437, China

## Abstract

**Purpose:**

To study the therapeutic effect of Quyu (QY) Shengxin (SX) decoction (QYSXD) in mice with dextran sulfate sodium- (DSS-) induced ulcerative colitis and to investigate the effects of QYSXD on the regulation of the receptor-interacting protein kinase 1 (RIP1)/receptor-interacting protein kinase 3 (RIP3)/nucleotide-binding oligomerization domain-like receptor family pyrin domain protein 3 (NLRP3) signaling pathway.

**Method:**

Thirty-six mice were randomly divided into the following 6 groups: the experimental group (QYSX group), the model group (DSS group), the positive control group (5-aminosalicylic acid (5-ASA) group), the control group, the first component group (QY group), and the second component group (SX group). Each group included 6 mice. Ulcerative colitis (UC) was induced in the mice by providing 3.5% DSS in drinking water. The mice were weighed every day to evaluate the disease activity index (DAI). After 7 days, the mice were sacrificed, and colonic tissues were obtained for colon length measurement. The morphological changes in the colon and the pathological scores of the mice in each group were observed by hematoxylin-eosin (HE) staining. The messenger ribonucleic acid (mRNA) and protein expression levels of RIP1, RIP3, dynamin-related protein 1 (Drp1), NLRP3, cysteinyl aspartate specific proteinase-1 (caspase-1), interleukin (IL)-1*β*, and IL-18 in the colon tissues of the mice in each group were detected and compared by real-time quantitative reverse transcription PCR (RT-qPCR) and enzyme-linked immunosorbent assay (ELISA). The levels of RIP1, RIP3, NLRP3, IL-1*β*, and IL-8 in the colonic mucosa were detected by ELISA. Western blotting was used to compare the protein expression of Drp1, caspase-1, mitochondrial fission protein 1 (FIS1), and mitophagy-associated protein light chain 3a/b (LC3a/b) among groups. The levels of reactive oxygen species (ROS) in the colonic mucosal cells were compared by immunofluorescence.

**Results:**

Compared with those in the DSS group, the mice with DSS-induced colitis in the QYSX group exhibited clearly higher body weights (*P* < 0.05) and DAI scores (*P* < 0.05). The colon lengths of the mice in the QYSX group were longer than those in the DSS group (*P* < 0.05), and the pathological score of the QYSX group was lower than that of the DSS group (*P* < 0.05). The RIP1, RIP3, Drp1, IL-1*β*, IL-18, and caspase-1 mRNA levels in the QYSX, 5-ASA, SX, and QY groups were significantly lower than those in the DSS group (*P* < 0.05), but there were no differences between the QYSX group and the 5-ASA group. The levels of RIP1, RIP3, NLRP3, IL-1*β*, and IL-18 in the QYSX group were lower than those in the DSS group (*P* < 0.01). The levels of Drp1, caspase-1, FIS1, and LC3a/b in the QYSX group and the 5-ASA group were lower than those in the DSS group (*P* < 0.05). The levels of ROS in the colonic mucosal cells in the QYSX, 5-ASA, and QY groups were lower than those in the DSS group (*P* < 0.05).

**Conclusion:**

QYSXD has certain therapeutic effects on DSS-induced colitis in mice and may be as effective as 5-ASA. QY and SX decoctions also have certain effects on colitis; however, these decoctions are not as beneficial as QYSXD. QYSXD may ameliorate colitis by inhibiting the expression of RIP1/RIP3/NLRP3 pathway-related proteins and reversing mitochondrial dysfunction to control inflammation.

## 1. Introduction

Ulcerative colitis (UC) is a chronic, nonspecific inflammatory disease with unclear pathogenesis that can affect the rectum, rectosigmoid colon, and colon and is characterized by congestion, erosion, and ulcers of the mucosa [1]. The main symptoms of UC are intestinal mucosal barrier dysfunction, intestinal bleeding, the presence of mucus in stool, and purulent haematochezia. Prolonged lack of treatment of UC can lead to the development of colon cancer in patients. Pathological features, including mucosal inflammation, crypt structure disorder, Paneth cell metaplasia, and occur in the absence of goblet cells [2, 3]. The pathogenesis of UC is related to a variety of factors, such as genetics (8–14% of patients have inflammatory bowel disease (IBD) familial inheritance), environment, diet, intestinal microorganisms, and immune abnormalities [4]. The morbidity of UC is increasing worldwide, and it is higher in developing countries, especially in China, than in developed countries [4]. The receptor-interacting protein kinase (RIPK) family is a group of threonine/serine protein kinases with relatively conserved kinase domains and different nonkinase domains that participate in physiological and pathological processes, including nonspecific immune responses and inflammation [5, 6]. The RIPK family causes cell necrosis and triggers inflammation by participating in the formation of necrotic complexes, and RIPK 1 (RIP1) and RIPK 3 (RIP3) are particularly sensitive to cell necrosis and inflammation [7]. RIP1 and RIP3 play important roles in IBD [8]. In humans, RIP1 gene defects may cause IBD. In mice and humans, RIP3 deficiency causes obvious immunodeficiencies and intestinal inflammation symptoms and may even cause enteritis-type bowel cancer [9–11]. One study has found that the levels of RIP1 and RIP3 are significantly increased in mice with dextran sulfate sodium- (DSS-) induced colitis and that the RIPK inhibitor necrostatin-1 can effectively inhibit the RIP1/RIP3 interaction, thereby inhibiting intestinal inflammation [12]. Clear elevations in nucleotide-binding oligomerization domain-like receptor family pyrin domain protein 3 (NLRP3), interleukin (IL)-1*β*, and IL-18 levels have been discovered in the colon mucosae and sera of UC patients and are directly involved in the occurrence of inflammation [13]. NLRP3 increases susceptibility to IBD [14]. In addition, susceptibility to UC is evidently decreased by DSS in mice with NLRP3 gene defects. Furthermore, mice with cysteinyl aspartate specific proteinase-1 (caspase-1) gene knockout have fewer UC symptoms after treatment with DSS, and the mechanism could be related to reduced secretion of IL-18 and IL-1*β* in the colonic mucosal tissue [15].

Exogenous bacteria, viral ribonucleic acid (RNA), tumor necrosis factor-*α* (TNF-*α*), and lipopolysaccharide (LPS) have been shown to stimulate RIP1 and RIP3 activity via the formation of the RIP1-RIP3 complex, facilitating the activation of the GTPase dynamin-related protein 1 (Drp1) and its translocation to mitochondria and thus inducing mitochondrial damage, reactive oxygen species (ROS) production, and NLRP3 inflammasome activation [16]. Caspase-1 released by NLRP3 inflammasomes can stimulate the production of IL-1*β* and IL-18, thereby causing inflammation. In addition, increases in IL-18 levels can directly lead to necrosis, apoptosis, and destruction of the mucosal barrier function of the colon in mouse goblet cells. Conversely, a decline in IL-18 can reduce or even eliminate enteritis [17].

Traditional Chinese medicine (TCM) has been used in China for thousands of years as a traditional and complementary approach for treating UC and has fewer side effects than standard medicine [18, 19]. A growing number of Chinese herbal medicines or extracts (*Astragalus membranaceus*, *peach kernels*, *Euphorbia humifusa*, etc.) have proven effective for addressing UC symptoms [20]. Quyu (QY) Shengxin (SX) decoction (QYSXD) is a TCM decoction used for the clinical treatment of UC; it is administered orally for patients with mild to moderate UC. The decoction is an empirical prescription from Professor Wang Zhenyi of the Shanghai University of TCM. QYSXD contains a herbal mixture that removes blood stasis (QY) and an herbal mixture that promotes tissue regeneration (SX). The former includes *Persicae semen*, *Ligusticum chuanxiong* Hort, *Kummerowia striata* (Thunb.) Schindl., and *Euphorbia humifusa* Willd., while the latter includes *Astragalus membranaceus* (Fisch.) Bunge, *Pseudostellaria heterophylla* (Miq.) Pax, *Atractylodes macrocephala* Koidz., and *Rehmannia glutinosa* (Gaertn.) Steud., which can clear heat, detoxify, and remove blood stasis.

The decoction can effectively improve symptoms in UC patients with mucus in stool, purulent hematochezia, diarrhea, and abdominal pain. However, its functional mechanism is still unclear. Studies have shown that *Astragalus* polysaccharide reduces NLRP3 inflammasome activation and IL-1*β* and IL-18 levels in DSS-induced colitis [21, 22]. The active ingredients of *Euphorbia humifusa* Willd. also exert anti-inflammatory effects [23]. *Rehmannia-*derived catalpol can relieve cellular inflammation and the related symptoms of colitis in rats by deregulating miR-132 [24]. In addition, one study on QYSXD revealed 41 bioactive substances that are related to inflammation and immunity [25]. QYSXD can reduce the abundance of Caudovirales in the intestinal flora of UC patients and reverse phage disorders [26]. Nevertheless, whether the effects of QY herbs or SX herbs are more important for the ameliorative effects of QYSXD on UC is unknown. In the current study, QYSXD was divided into QY decoction (QYD) and SX decoction (SXD) to investigate the mechanism of this TCM treatment.

On the basis of previous evidence, we hypothesized that the QYSXD agent may be able to treat UC by regulating the RIP1/RIP3/NLRP3 signaling pathway. This experiment was designed to explore the therapeutic effects of QYSXD and its decomposed components on DSS-induced UC.

## 2. Materials and Methods

### 2.1. Reagents

All herbs in QYSXD and the decomposed components were purchased from Tianyin Tianjiang Pharmaceutical Co., Ltd. (Jiangyin, Jiangsu Province, China) and conformed to the standards of the Chinese Pharmacopoeia. The decoction included 45 g of *Astragalus membranaceus* (Fisch.) Bunge; 30 g of *Pseudostellaria heterophylla* (Miq.) Pax; 15 g each of *Rehmannia glutinosa* (Gaertn.) Steud., *Atractylodes macrocephala* Koidz., *Persicae semen*, and *Ligusticum chuanxiong* Hort; and 20 g each of *Euphorbia humifusa* Willd. and *Kummerowia striata* (Thunb.) Schindl. DSS (MW 36,000–50,000 Da) was obtained from MP Biomedicals (Irvine, California, USA). LPS was obtained from Sigma-Aldrich Corp. (St. Louis, MO, USA). A PrimeScriptRT Reagent Kit with gDNA Eraser (Perfect Real Time) and SYBR® Premix Ex Taq™ II were purchased from TaKaRa Bio Inc. TRIzol was obtained from Invitrogen Scientific Co., Ltd. (Waltham, Massachusetts, USA). Antibodies against Drp1, FIS1, caspase-1, light chain 3 (LC3), and *β*-actin were purchased from Servicebio Corp. (Wuhan, China). Enzyme-linked immunosorbent assay (ELISA) kits for RIP1, RIP3, NLRP3, IL-1*β*, and IL-18 were purchased from Sigma-Aldrich Corp. Dihydroethidium (DHE) for ROS analysis and 4′,6-diamidino-2-phenylindole (DAPI) were purchased from Servicebio Corp. (Wuhan, China).

### 2.2. Animals and Experimental Design

Thirty-eight specific pathogen-free (SPF) male BALB/*c* mice weighing 14.5 g to 17.2 g were purchased from the Animal Experimental Center of Shanghai University of TCM. After adaptive feeding for 2 weeks in a clean laboratory, 7 mice were grouped into the control group, and the rest of the mice were administered 3.5% DSS solution in distilled water for 7 days. One mouse from the control group and one DSS-treated mouse were randomly selected, their colons were harvested, and the colon tissues were stained with hematoxylin-eosin (HE) to verify the successful establishment of the UC model. The remaining thirty DSS-treated mice were divided into 5 groups: the DSS group, the 5-aminosalicylic acid (5-ASA) group, the QYSX group, the SX group, and the QY group.

Drug treatment began on the second day after the successful induction of UC. According to the drug dose conversion formula for humans and mice, the gastric perfusion doses were 26.25, 15.75, 10.5, and 0.6 g/kg in the QYSX, SX, QY, and 5-ASA groups, respectively. All groups except for the control group were treated by constant gavage for 7 days, and body weight, hematochezia, and stool blood data were recorded every day.

The TCM decoction for the experiment was made using a water extraction and alcohol precipitation method. The raw medicine was boiled twice, and the resulting decoctions were mixed and concentrated to 10 g/ml. Then, 95% ethanol was added to the concentrated decoction to a final concentration of 75%, and the solution was left to stand for 24 hours to allow the ethanol to evaporate completely. The decoction was filtered, and impurities were removed by repeating the above process twice. The treated decoction was diluted with water into a TCM solution with a crude drug content of 6 g/ml. The finished decoctions were prepared in the preparation room of Shanghai Baoshan Integrated Traditional Chinese and Western Medicine Hospital, and mesalazine granules (procured in Shanghai) were used as the positive control.

### 2.3. Disease Activity Index (DAI) Scores

Mouse disease was scored using the standard DAI scale [27] (as shown in Table 1) on the basis of daily weights, stool consistency, and the presence of blood in stool during modeling.

A fecal occult blood test (with colloidal gold) was used to detect fecal occult blood in mice. In brief, 1.0 ml of saline was added to a rubber EP tube containing one piece of crushed mouse feces and then mixed; the concentration was approximately 3–5%. The fecal suspension (0.5 ml) was dripped into the S area of a test card with a straw, and the test card was set aside to develop for 5–10 min. An obvious red band in the detection area (T) and a red band in the control area (C) indicated a positive result, a red band in the T region and a light red band in the C region represented a weakly positive result, and a band only in the C region indicated a negative result.

### 2.4. Histological Score

The pathological scores included the degree of inflammatory cell infiltration, mucosal injury, and intestinal mucous gland crypt injury. Pathological changes in the grouped mouse pathological sections were observed by microscopy after HE staining. The pathological scores are outlined in Table 2.

### 2.5. Detection of RIP1, RIP3, Drp1, NLRP3, IL-1*β*, IL-18, and Caspase-1 mRNA in Colon Tissue by RT-qPCR

RNA was extracted by the TRIzol method and tested using a Qubit 2.0 and agarose gel electrophoresis. The reaction system included 1 *μ*g of total RNA per 20 *μ*l of the reverse transcription system. After removing genomic DNA, the reaction system was composed of the following components: 5 × gDNA Eraser Buffer, 2 *µ*l of gDNA Eraser, 1 *µ*l of total RNA, and 1 *μ*g of RNase-free ddH_2_O. Reverse transcription master mix was prepared in a new RNase-free centrifuge tube after the reaction mixture was incubated for 2 min at 42°C and transferred to ice. The reaction solution (10 *μ*l) was added to the samples after genomic DNA removal. cDNA was obtained by mixing the tubes at 37°C for 15 min and then reacting them at 85°C for 5 seconds. The cDNA samples were diluted twice and used as templates for amplification. Each sample was added to 3 wells and run in a Quant Studio 6 Flex RT-qPCR system. The cycle thresholds (Ct) of RIP1, RIP3, NLRP3, and IL-1*β* mRNA were determined and analyzed by the ΔΔCt method (ΔΔCt = ΔCt − ΔCt blank value, ΔCt = Ct target gene-Ct internal reference). The relative expression of each gene was calculated according to the 2^−*ΔΔCt*^ formula. The primers are shown in Table 3.

### 2.6. Measurement of RIP1, RIP3, NLRP3, IL-1*β*, and IL-8 Levels in Colon Tissues by ELISA

Protein was extracted from the tissue samples following the protocols provided with the ELISA kits, and the corresponding protein levels were determined by the double-antibody Sandwich method. A purified mouse protein antibody was coated on a microwell plate and was used as the solid-phase antibody. After the protein was added to the monoclonal antibody-coated microwell plate and bound to an HRP-conjugated antibody, an antibody-antigen-enzyme-labeled antibody complex was formed. The reaction was developed by adding the substrate TMB after thorough washing. TMB becomes blue under the catalysis of the enzyme HRP and converts to a yellow color under the action of acid. The absorbance (optical density (OD) value) was measured with an ELISA reader at a wavelength of 450 nm, and the concentration of mouse protein was calculated using a standard curve.

### 2.7. Measurement of Drp1, Mitochondrial Fission Protein 1 (FIS1), Caspase-1, LC3a/b, and GAPDH Expression in Colon Tissues by Western Blotting

Following the description of the protein extraction kit, the total protein was extracted from colon tissues. The protein concentration was determined by the bicinchoninic acid (BCA) method. The proteins were electrophoretically separated by SDS-PAGE using a gel with the appropriate percentage of SDS and wet-transferred to nitrocellulose membranes according to the relative molecular weights of the Drp1, FIS1, caspase 1, LC3a/b, and GAPDH proteins. Then, 5% defatted milk powder was added to block the membranes at room temperature for 2 hours. Diluted primary antibodies were added, and the membranes were incubated overnight at 4°C and washed 3 times with TBS-T. The corresponding secondary antibodies were added, and the membranes were incubated at room temperature for 2 hours and then washed 3 times with TBS-T. Images of the membranes were obtained after the development of chemiluminescence. Greyscale analysis of the reaction bands in the images was conducted with Alpha software, and the relative expression of each protein was determined according to the following equation: target protein band density/GAPDH band density.

### 2.8. Measurements of ROS Levels in Colon Tissue by Immunofluorescence Assay on Frozen Sections

Sections of colon tissue were frozen and sectioned. The frozen slides were restored to room temperature, the obvious liquid was removed, and the tissue of interest was marked with a liquid blocker pen. Spontaneous fluorescence quenching reagent was added, and the sections were incubated for 5 min. The samples were washed in running tap water for 10 min. ROS staining solution was added to the marked area, and the samples were incubated at 37°C for 30 min and stored in the dark. DAPI was used to counterstain the nuclei, and the samples were washed three times with PBS (pH 7.4) on a rocker device for 5 min each. The small amount of liquid was then removed, and the samples were covered with coverslips and mounted with an antifade mounting medium. Fluorescence microscopy was performed for visualization and image collection. DAPI appeared blue at a UV excitation wavelength of 330–380 nm and an emission wavelength of 420 nm; FITC appeared green at an excitation wavelength of 465–495 nm and an emission wavelength of 515–555 nm; CY3 appeared red at an excitation wavelength of 510–560 nm and an emission wavelength of 590 nm. The nuclei were labeled blue by DAPI. ROS-positive cells were labeled red by fluorescein.

### 2.9. Statistical Analysis

All the data are expressed as the mean ± standard deviation. SPSS 18.0 statistical software was used to analyze the differences among groups by one-way analysis of variance (ANOVA). *P* < 0.05 was considered to indicate statistical significance.

## 3. Results

### 3.1. QYSXD Increased Body Weight and Reduced DAI Scores and Colon Tissue Lengths

**Figure 1:**
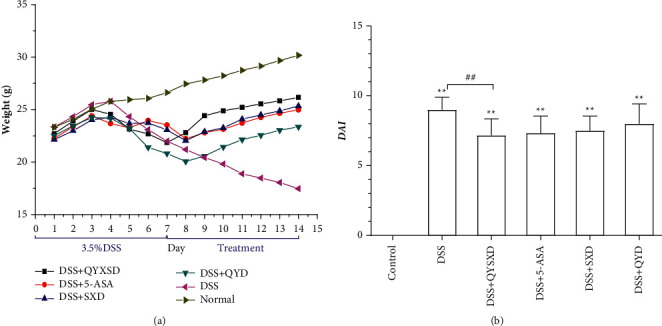
Effects of QYSXD, QYD, and SXD on mouse body weight (a) and DAI (b). The mice in the QYSX, QY, SX, and 5-ASA groups had markedly greater body weights than the mice in the DSS group (*P* < 0.05). Compared with those in the control group, the body weights of mice in the QYSX, QY, SX, and 5-ASA groups were all lower (*P* < 0.05). DAI score: ^##^*P* < 0.01.

As shown in Figure 1, the body weights of the mice tended to be decreased on the 4^th^ day after 3.5% DSS administration. The weights of the mice in each group were regained after drug intervention. The group with the most obvious effect was that treated with QYSXD (*P* < 0.05). The DAI score exhibited the most significant decrease in the QYSX group; the score in this group was lower than those in the DSS group (*P* < 0.01), the SX group (*P* < 0.05), and the QY group (*P* < 0.05); however, there was no difference between the QYSX group and the 5-ASA group.

**Figure 2:**
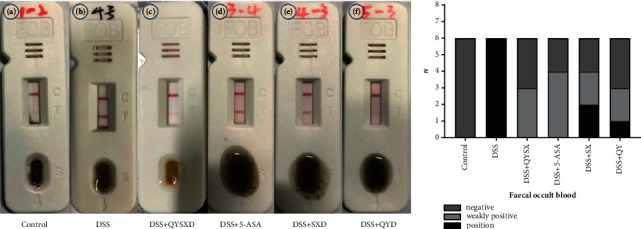
The control group was negative for fecal occult blood (a), while the DSS group was positive (b). The QYSX group (c), 5-ASA group (d), SX group (e), and QY group (f) were weakly positive for fecal occult blood.

Figure 2 shows that the mice in the control group were negative for fecal occult blood. All the mice in the DSS group were positive for fecal occult blood. In contrast, 3 mice in the QYSX group were weakly positive, and the other 3 were negative; 4 mice in the 5-ASA group were weakly positive, and the other 2 were negative; 2 mice in the SX group were positive, 2 were weakly positive, and 2 were negative; and 1 mouse in the QY group was positive, 2 were weakly positive, and the other 3 were negative.

**Figure 3:**
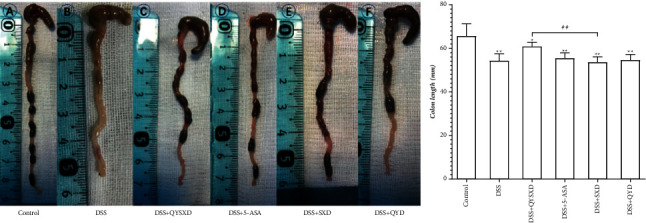
The colon lengths of the mice in the QYSX group were clearly longer than those of the mice in the DSS group (^##^*P* < 0.01) and the SX group (^##^*P* < 0.01). Compared with that in the control group, the colon length was shorter in the QYSX, QY, SX, and 5-ASA groups (^*∗∗*^*P* < 0.01). (a) DSS group (b). Control group (c), QYSX group (d) 5-ASA group (e) SX group, and QY group (f).

The DSS group (a) exhibited the shortest colon length, with an average value of 54.33 mm, while the control group (b) exhibited the longest colon length, with an average value of 65.67 mm (see Figure 3). The QYSX group (c) exhibited the longest colon length among the treatment groups, with an average value of 60.83 mm. The average colon length in the 5-ASA group (d) was 55.5 mm, the average colon length in the SX group (e) was 53.67 mm, and the average colon length in the QY group (f) was 54.67 mm. The colon length in the QYSX group was shorter than that in the control group and longer than that in the DSS group (*P* < 0.05). Compared with that in the QYSX group, the colon length in the SX group was significantly shorter (*P* < 0.01). There was no difference between the QY group and the DSS group (*P* > 0.05).

### 3.2. QYSXD Alleviated Pathological Injury in Colon Tissue

**Figure 4:**
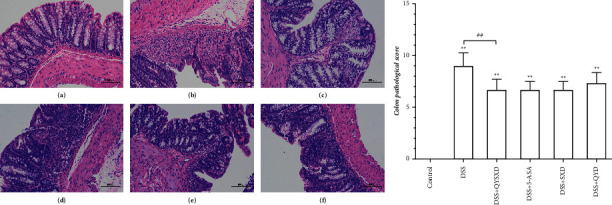
Pathological examination revealed that the mucosa of the colon in the control group was intact (a). In the DSS group, the mucosa was mostly absent, and inflammatory infiltration was obvious (b). In the QYSX group, the mucosa was intact, with slight inflammatory infiltration (c). The mucosa in the 5-ASA group was more disorganized than that in the QYSX group, and inflammatory infiltration was more obvious (d). In the SX (e) and QY (f) groups, some mucosal tissue was absent, and inflammatory cells infiltrated into the mucosa and muscularis layer. The QYSX group had the lowest pathological score, which was different from that of the DSS group (^##^*P* < 0.01).

A significant difference in the pathological score (see Figure 4) was observed between the QYSX group and the DSS group (*P* < 0.01). Inflammatory cells clearly appeared in the colonic mucosae of DSS-treated mice, and the numbers of epithelial cells decreased, with disordered or absent local gland structures, as shown by HE staining of the pathological sections. The colonic mucosal epithelial cells of the mice in the QYSX group remained relatively intact, and a small amount of lymphocyte infiltration was visible in the lamina propria.

### 3.3. Effects of QYSXD on RIP1, RIP3, NLRP3, Caspase-1, IL-1*β*, and IL-18 mRNA Expression in Colon Tissue

**Figure 5:**
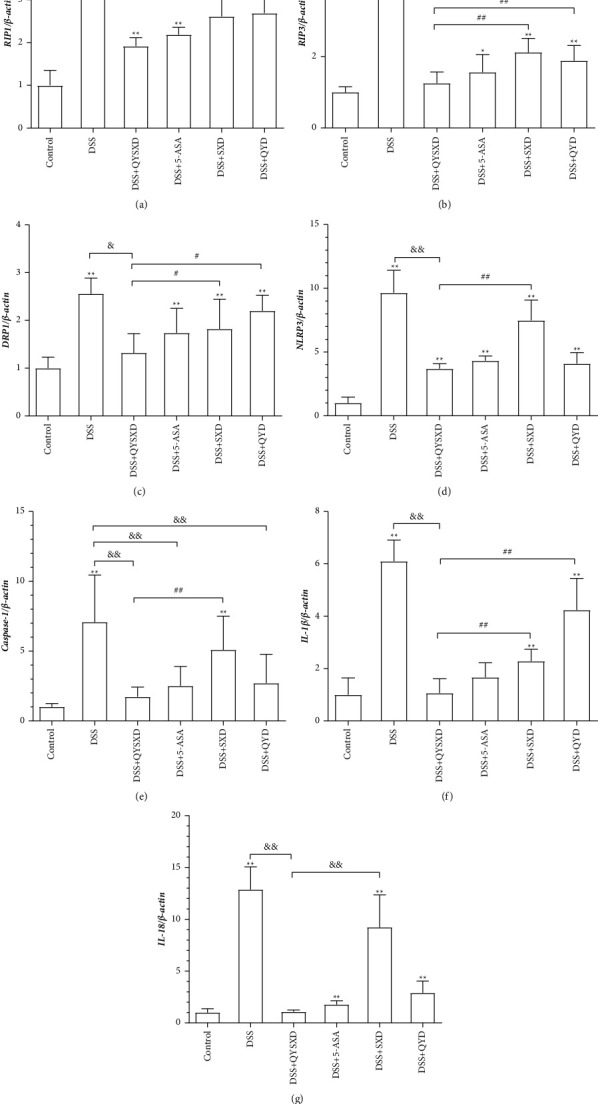
mRNA expression of RIP1 (a), RIP3 (b), Drp1 (c), NLRP3 (d), caspase-1 (e), IL-1*β* (f), and IL-18 (g) in each group. The mRNA expression was significantly elevated in the DSS group and was the lowest in the control group. Compared with the DSS group, the QYSX group presented significantly lower mRNA levels of RIP1, RIP3, Drp1, NLRP3, caspase-1, IL-1*β*, and IL-18 (*P* < 0.05). The mRNA expression of RIP1 and Drp1 was higher in the SX group (^#^*P* < 0.01) and QY group (^##^*P* < 0.01) than that in the QYSX group. Compared with those in the QYSX group, the RIP3 and IL-1*β* mRNA levels were higher in the SX group (^##^*P* < 0.01) and QY group (^##^*P* < 0.01). There were significant differences in NLRP3 mRNA expression between the QYSX group and the SX group (^##^*P* < 0.01). The mRNA expression of caspase-1 in the QYSX group was lower than that in the SX group (^##^*P* < 0.01), and there were significant differences among the 5-ASA group, the QY group, and the DSS group (^&&^*P* < 0.01). The mRNA expression of IL-18 in the QYSX group was lower than that in the SX group (^&&^*P* < 0.01).

A series of factors were modified by 3.5% DSS-induced colitis. Increased expression of RIP1, RIP3, NLRP3, the NLRP3 substrate caspase-1, IL-1*β*, and IL-18 was observed (Figure 5). The expression of all these molecules apparently decreased after QYSXD intervention, resulting in significantly lower expression in the QYSX group than in the DSS group (*P* < 0.05). In the QYSX group, the RIP1, RIP3, and IL-1*β* levels were lower than those in the SX and QY groups, while the NLRP3, IL-18, and caspase-1 levels were lower than those in the SX group. There were no significant differences between the QY and 5-ASA groups.

### 3.4. QYSXD Decreased the Expression of RIP1, RIP3, NLRP3, IL-1*β*, and IL-18 in Colon Tissue

**Figure 6:**
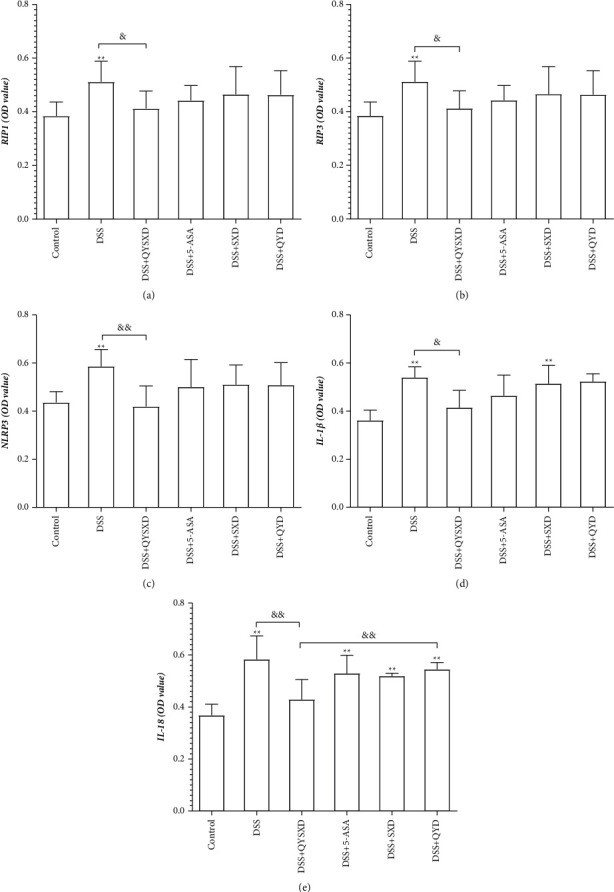
The levels of RIP1 (a) and RIP3 (b) in the DSS group were significantly higher than those in the control group (*P* < 0.001), and those in the QYSX group were clearly lower than those in the DSS group (*P* < 0.05). NLRP3 expression (c) was significantly higher in the DSS group than that in the control group (^*∗∗*^*P* < 0.001) and significantly lower in the QYSX group than that in the DSS group (^&^*P* < 0.01). The IL-1*β* (d) levels in the DSS group, QY group, and SX group were significantly higher than those in the control group (^*∗∗*^*P* < 0.001), and the IL-1*β* level in the QYSX group was significantly lower than that in the DSS group (*P* < 0.05). The IL-18 levels (e) in the DSS group, 5-ASA group, SX group, and QY group were significantly higher than those in the control group (^*∗∗*^*P* < 0.001), and the IL-18 level in the QY group was significantly higher than that in the QYSX group (^&&^*P* < 0.01).

Indicating from Figure 6, QYSXD was able to reduce the expression of RIP1, RIP3, NLRP3, IL-1*β*, and IL-18. Similar to QYSXD, 5-ASA also decreased the expression of RIP1, RIP3, NLRP3, IL-1*β*, and IL-18, but the effects were relatively minor. Moreover, the QY group and SX group exhibited reduced protein expression to some extent, but no significant differences were observed.

### 3.5. Effect of QYSXD on Drp1, Caspase-1, FIS1, and LC3a/b Expression in Colon Tissue

**Figure 7:**
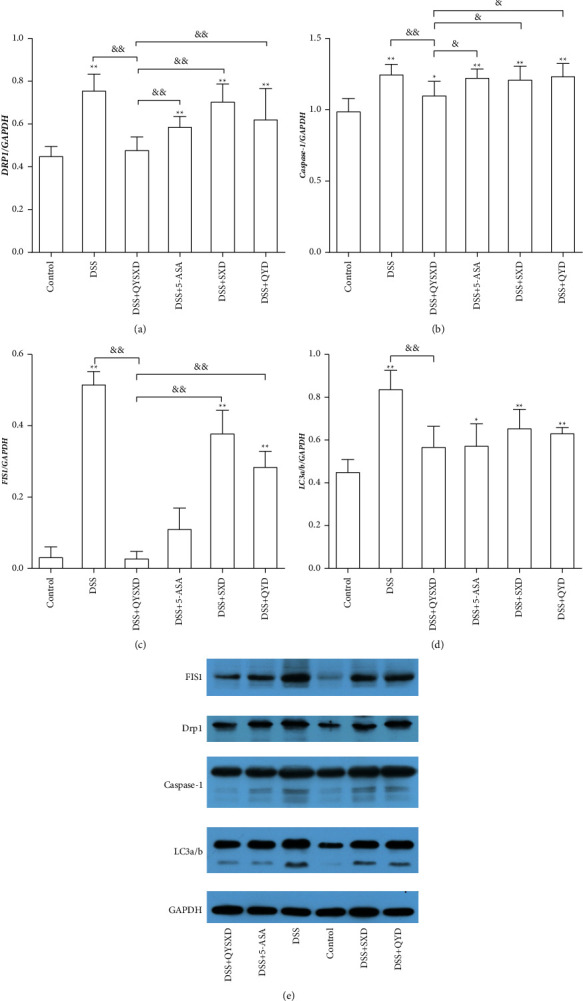
The levels of Drp1 (a) in the DSS group, 5-ASA group, SX group, and QY group were significantly higher than those in the control group (^*∗∗*^*P* < 0.01). The level of Drp1 in the QYSX group was significantly lower than that in the DSS group, 5-ASA group, SX group, and QY group. The level of Drp1 in the 5-ASA group was also lower than that in the DSS group (*P* < 0.05). The levels of caspase-1 (b) in the DSS group, QYSX group, 5-ASA group, SX group, and QY group were significantly higher than those in the control group. The caspase-1 level in the QYSX group was significantly lower than that in the DSS group, 5-ASA group (^&^*P* < 0.05), SX group (^&^*P* < 0.05), and QY group (^&^*P* < 0.05). The levels of FIS1 (c) in the DSS group, SX group, and QY group were significantly higher than those in the control group (^*∗∗*^*P* < 0.01). The FIS1 level in the QYSX group was significantly lower than those in the DSS group, SX group, and QY group (^&&^*P* < 0.01), and the FIS1 level in the 5-ASA group was also lower than that in the DSS group (^&&^*P* < 0.01). The level of LC3a/b (d) in the DSS group, 5-ASA group, SX group, and QY group was significantly higher than that in the control group. The value in the QYSX group was significantly lower than that in the DSS group (^&^*P* < 0.01).

QYSX effectively reduced the expression of FIS1 (*P* < 0.01) and LC3a/b (*P* < 0.01) in colon tissue (Figure 7). A significant difference was observed between the QYSX and SX groups (*P* < 0.01) and between the QYSX and QY groups (*P* < 0.01) in the regulation of FIS1; however, compared with the QYSX group, the control group showed only a slight difference (*P* > 0.05). With regard to the regulation of LC3a/b, the LC3a/b level in the QYSX group was clearly lower than that in the DSS group (*P* < 0.01) and higher than that in the control group (*P* < 0.05). The Western blot results showed that QYSX effectively reduced the expression of Drp1 but did not affect caspase-1 expression.

### 3.6. QYSXD Reduced ROS Production in Colon Tissue

**Figure 8:**
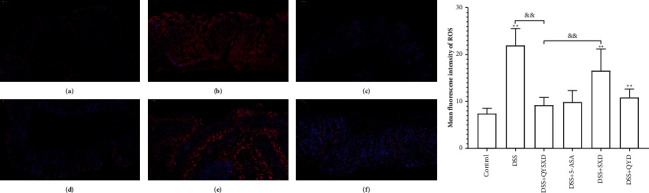
The fluorescence of ROS was the weakest in the control group (a). The fluorescence was significantly stronger in the DSS group than in the other groups (b). The red fluorescence in the QYSX group (c) was similar to that in the 5-ASA group (d). Among the treatment groups, the SX group exhibited the strongest ROS fluorescence (e), and the ROS fluorescence in the SX group was significantly stronger than that in the QY group (f). The fluorescence values of ROS (a) in the DSS group, SX group, and QY group were significantly higher than those in the control group (^*∗∗*^*P* < 0.01). The fluorescence value of ROS in the QYSX group was significantly lower than that in the SX group (^&&^*P* < 0.01), and there was no significant difference between the 5-ASA group and the QY group (*P* < 0.05).

Figure 8 shows that the QYSX group exhibited the weakest ROS fluorescence in the treatment group. The fluorescence of the QY group and SX group was more obvious than that of the 5-ASA group in the images. The levels of intracellular ROS were significantly lower in the QYSX, 5-ASA, and QY groups than in the DSS group (*P* < 0.01). However, the SX group exhibited a minor difference compared with the DSS group, and the ROS level in the SX group was not clearly different from the levels in the QYSX group and the SX group. In the immunofluorescence images, ROS-positive staining was more intense in the DSS group than in the other groups. ROS fluorescence in the QYSX group was weaker than that in the other treatment groups, and ROS fluorescence in the QY group was weaker than that in the SX group.

## 4. Discussion

The main pathological manifestations of UC are infiltration of lymphocytes, plasma cells, eosinophils, and neutrophils in the mucosa and submucosa; crypts that can form abscesses; intestinal epithelial cell necrosis that can result in ulceration [28]; and a lack of an intestinal mucosal barrier, which increases the permeability of the intestinal mucosa [29]. Intestinal epithelial cells, goblet cells, Paneth cells, and the mucus layer, which form the first intestinal barrier, defend intestinal health [30]. Regulating excessive cell necrosis and reducing the occurrence of inflammation may be an effective approach to UC treatment.

DSS-induced colitis is the most widely used, replicable, and classic UC animal model [31, 32]. The appearance of induced colitis in mice includes congestion, edema, erosion, and even intestinal mucosal ulcers and colon length shortening. The symptoms are loose stool and hematochezia. In the current study, after DSS removal, the symptoms in BALB/*c* mice disappeared in 2 weeks, and the pathological changes in the intestinal tissue and related cytokines recovered in approximately 4 weeks. This study showed that after one week of QYSXD treatment, the body weight, hematochezia, and diarrhea (DAIs) of DSS-induced mice were effectively improved; the inflammatory infiltration of the mucosal and muscularis layers was alleviated; and mucosal ulcers were partially healed, showing improvement of colon pathology. Additionally, the pathological score was elevated in the DSS group, and the therapeutic effect of QYSXD was similar to that of 5-ASA, which indicated the successful effect of QYSXD on DSS-induced colitis.

The RIP1 and RIP3 levels in mice with DSS-induced colitis were significantly higher than those in control mice. RIP1 and RIP3 are related proteins that participate in programmed cell death [33]. Abnormal increases in RIP1 and RIP3 expression can cause cell death, immune abnormalities, or inflammation [34]. RIP1 is also a key molecule that maintains intestinal epithelial barrier function [9]. IL-1*β* expression and DSS-induced intestinal inflammation can be reduced by blocking the signaling pathway related to RIP3 [35]. Previous research [33] has also confirmed that RIP1/RIP3 can regulate cell necrosis and the expression of downstream inflammatory factors by mediating the tumor necrosis factor receptor 1 (TNFR1), Toll, and NLR signaling pathways, which leads to cellular inflammation. GSK2982772, a clinical candidate drug developed by Harris et al. [36], can inhibit the activity of RIP1, protect against UC, slow weight loss, alleviate disease severity, and reduce intestinal inflammatory cell infiltration in mice treated with DSS. The current study also revealed that RIP1 and RIP3 levels were significantly lower in the group with QYSXD intervention than in the control group. Among UC patients and mice with DSS-induced colitis, the numbers of NLRP3 inflammatory complexes are increased significantly. The production of mature IL-1*β* and IL-18 can be stimulated after NLRP3 inflammatory complex activation [37, 38]. However, it has also been found that the IL-1*β* and IL-18 produced by NLRP3 inflammatory complexes have protective effects against colitis, although the mechanism is still unclear [39]. In this study, clear increases in IL-1*β* and IL-18 were observed in the colonic mucosae of mice with DSS-induced colitis. QYSXD, 5-ASA, QYD, and SXD treatment tended to decrease the levels of IL-1*β*, IL-18, NLRP3, and caspase-1. These findings suggest that QYSXD is an effective anti-inflammatory agent that inhibits the expression of RIP1 and RIP3 and controls downstream inflammatory factors. However, QYD and SXD, the separate components of QYSXD, were not as effective in regulating the above cytokines as the QYSX decoction. Similar studies have also found that the effect of QYSXD is better than that of the separate decoction components [40].

QYSXD clearly reduced Drp1, FIS1, and LC3a/b expression. Drp1, FIS1, and LC3a/b are the key proteins involved in mitochondrial autophagy. Obvious mitochondrial dysfunction and mitochondrial autophagy occur in the context of DSS-induced colitis [41]. However, restoration of mitochondrial function can reduce colon inflammation to a certain extent [42, 43]. The main cause of mitochondrial dysfunction is the intracellular production of large amounts of ROS that translocate to the mitochondria [44]. The expression of the mitochondrial autophagy marker proteins Drp1, FIS1, and LC3a/b was significantly elevated in the colonic mucosae of DSS-treated mice; however, the expression of these proteins was dramatically decreased, and the immunofluorescence of ROS in tissue samples was also clearly reduced, in the QYSX group compared with the DSS group.

Different TCM treatments must be used for different diseases based on the patient's symptoms, tongue coating, and pulse; the appropriate treatment can be selected by referring to TCM theory and clinical experience to determine the compatibility of a variety of Chinese herbs. TCM treatment combines several basic treatments, indicating that a single treatment is unlikely to have an obvious effect. In this study, the component decoctions QYD and SXD were observed to have certain anti-inflammatory effects; however, the effects were weaker than those of QYSXD, which indicated that the Chinese herbal medicine components exerted synergistic effects rather than simple additive effects.

This study preliminarily confirms that the ameliorative effects of QYSXD on UC may occur via the reversal of mitochondrial autophagy through the RIP1/RIP3/NLRP3 signaling pathway and the regulation of IL-1*β* and IL-18 to repair the barrier function of the intestinal mucosa. Further studies on the mechanism by which QYSXD acts on TNFR1 and other related pathway factors are needed to verify the roles of the related signaling pathways in effects on inflammatory cells and to identify the exact therapeutic mechanism of QYSXD.

## 5. Conclusions

QYSXD had a clear therapeutic effect on DSS-induced colitis in mice. The decoction may have exerted an anti-inflammatory effect by regulating the RIP1/RIP3/NLRP3 signaling pathway. In addition, QYSXD was more effective than the individual component decoctions—QYD and SXD—and was as effective as 5-ASA for treating enteritis. A further validation study of the signaling pathway is needed to confirm the exact mechanism of action of QYSXD.

## Figures and Tables

**Table 1:** DAI scoring criteria.

Score	Weight loss %	Stool consistency	Blood in stool
0	0	Normal	Normal
1	1–5	Loose	Positive (+)
2	5–10	Loose	Positive (++)
3	10–15	Pulpy	Positive (+++)
4	≥15	Watery	Positive (+++++)

*Note*. The three scores were added to calculate the final score.

**Table 2:** Histopathological scoring criteria.

Item	Score	Description
Inflammatory cell infiltration	0	Scattered inflammatory cells in the lamina propria
	1	Increased numbers of inflammatory cells in the lamina propria
	2	Inflammatory cell accumulation and invasion in the submucosa
	3	Inflammatory cell wall penetration

Mucosal injury	0	Clear mucosal boundaries, normal morphology
	1	Discrete epithelial injury
	2	Superficial mucosal corrosion or focal ulceration
	3	Possible expansion of mucosal injury to the deep intestinal wall

Crypt injury	0	Normal crypt morphology
	1	Damage to the basal 1/3
	2	Damage to the basal 2/3
	3	Only the epithelial surface is intact
	4	Complete disappearance of glandular crypts

**Table 3:** Primer sequences.

	Forward primer	Reverse primer
RIP1	5′-CCTTCTTGCCCAGGAGAATGA-3′	5′-CTCTGAGGCGATCTGACGAC-3′
RIP3	5′-CGTAGACGTCGGGTTTCCAG-3′	5′-ACCAGTAGGCCATAACTTGACA-3′
Drp1	5′-ACACGATTGAAGGAACCGCA-3′	5′-CGCTTAATCTGACGTTTGACC-3′
NLRP3	5′-TCTGCACCCGGACTGTAAAC-3′	5′-CACCCAACTGTAGGCTCTGC-3′
IL-1*β*	5′-ATAGGCTCATCTGGGATCCTCT-3′	5′-ACAGGTCATTCTCATCACTGTCAA-3′
IL-18	5′- ACAGGCCTGACATCTTCTGC-3′	5′- ATTGTTCCTGGGCCAAGAGG-3′
Caspase-1	5′- TGCCCAGAGCACAAGACTTC-3′	5′- TCCTTGTTTCTCTCCACGGC-3′
*β*-Actin	5′-GACGGCCAGGTCATCACTATTG-3′	5′-CCACAGGATTCCATACCCAAGA-3′

## Data Availability

All the data generated or analyzed in this study are included in the article.
